# Ten Years of African Swine Fever in Ukraine: An Endemic Form of the Disease in the Wild Boar Population as a Threat to Domestic Pig Production

**DOI:** 10.3390/pathogens11121459

**Published:** 2022-12-02

**Authors:** Hanna Omelchenko, Natalia O. Avramenko, Maksym O. Petrenko, Jarosław Wojciechowski, Zygmunt Pejsak, Grzegorz Woźniakowski

**Affiliations:** 1 Department of Normal and Pathological Anatomy and Physiology of Animals, Poltava State Agrarian University, 36-0036 Poltava, Ukraine; 2 Private Veterinary Practice, Grabowa 3, 86-300 Grudziadz, Poland; 3 Department of Infectious and Parasitic Diseases, The University Centre of Veterinary Medicine JU-AU, 31-120 Krakow, Poland; 4 Department of Infectious and Invasive Diseases and Veterinary Administration, Institute of Veterinary Medicine, Faculty of Biological and Veterinary Sciences, Nicolaus Copernicus University, 87-100 Toruń, Poland

**Keywords:** African swine fever, Ukraine, wild boar, domestic pigs, spread, epidemiological analysis

## Abstract

(1) Background: African swine fever (ASF) has been present in Ukraine for more than ten years (2012–2022). The purpose of our study was to perform a retrospective analysis of the spread of ASF to assess the role of wild boar in the epizootic expansion in Ukraine. (2) Methods: Statistical materials were collected and the epizootic situation of ASF from 2012 to 2022 was examined. The potential sources of the African swine fever virus (ASFV) and transmission factors were analysed. The main factors exerting negative impacts on domestic pig production were also analysed. (3) Results: Consequently, from the results of the retrospective analysis of ASF outbreaks in Ukraine, the probability ratio of ASF outbreaks in the wild boar and domestic pig populations was determined. The data show a direct relationship between ASF outbreaks among wild boar and domestic pigs with the observed decay of wild boar outbreaks across the entire territory of Ukraine. At the same time, an increase in the number of wild boars has been observed in the Mykolaiv region, with a parallel spillover of outbreaks in domestic pigs. (4) Conclusions: The epidemiological situation observed for ASF in the wild boar population may suggest an endemic form of the disease. This may further complicate eradication programs and the protection of domestic pig farms from ASF outbreaks. An additional and major reason to control the ASF epizootic is the continuing military Russian offensive in Ukraine.

## 1. Introduction

African swine fever (ASF) is a viral haemorrhagic disease with extremely high mortality in domestic pigs and Eurasian wild boar. During the last decade, ASF has appeared in several European and Asian countries and is now spreading in unprecedented numbers [1,2,3,4,5,6,7,8,9,10,11,12,13,14,15,16,17,18,19,20,21,22,23]. As an endemic disease in Europe, ASF was registered in 1978 on the Italian island of Sardinia [24], and since 2007 (the first report in Georgia), the disease has been registered in many Eastern European countries. One possible cause of the disease spreading to Georgia was the use of pigswill from ships carrying pigs and pig products contaminated with the African swine fever virus (ASFV), from where the disease spread further throughout the region [18,19,25]. 

ASF, which re-entered Europe in 2007 from Georgia, quickly hit neighbouring countries. It has caused serious economic damage to the Russian Federation and spread to the northern and western regions, including Ukraine (2012, 2014) and Belarus (2013). In early 2014, dead wild boar infected with the ASFV were found in Lithuania and Poland. Later, several outbreaks were reported in the European Union, affecting domestic pigs and wild boars in Latvia, Lithuania, and Poland, as well as wild boars in Estonia, causing serious problems for the EU pig sector [14,16]. In some regions of the Russian Federation, ASF is an endemic disease with which populations of domestic pigs and wild boar are widely affected. In contrast, in the affected countries of the Eastern European Union (EU), where the ASF epidemic is now present, the disease mainly affects wild boar populations in restricted areas and, to a much lesser extent, domestic pigs [26]. Unpredictably, the disease occurred in wild boar and domestic pig populations in Italy in 2022 [10]. The number of private pig farms is the largest in the western regions of Ukraine, and the spread of ASF to the west could lead to contamination of the territory near the EU border, which is difficult to control. In areas of the Russian Federation where wild boar density is high, the risk of ASF may be much higher. The majority of ASF outbreaks occur in the backyard sector of pig farming due to the neglect of biosecurity conditions [9,27,28,29].

The ASFV is a large icosahedral DNA virus that replicates predominantly in the cytoplasm of infected cells [30,31]. Recent ASF outbreaks in Kenya and Uganda have shown the coexistence of different ASFV genotypes in *Ornithodoros*, soft ticks. It is clear that *Ornithodoros* ticks play a major role in ASFV diversity as a most important biological vector of the virus in Africa [26]. The most characteristic clinical signs among infected pigs include high fever (42.0–42.5 °C), listlessness, lack of appetite, bloody diarrhoea, and vomiting. In addition, severe skin haemorrhages have been observed in affected animals, especially on the medial and lateral sides of the auricle, forelimbs, wrist, snout, scrotum, and mammary glands [13]. Pigs that survive can be permanently infected with the ASFV for months, so they can play an important role in virus transmission and the expansion of the disease, thus complicating attempts at its eradication [32,33]. 

Post-mortem lesions include darkening and enlargement of the spleen, severe haemorrhages to the mesenteric and gastrointestinal lymph nodes, and haemorrhagic enteritis [13]. ASF causes fatal haemorrhagic disease that can be transmitted by direct contact with infected animals and their secretions or indirect contact with contaminated fomites [3,34,35]. To minimise the risk of pathogen transmission, feed from areas affected by ASF should not be used in pigs [9,35,36,37]. On EU territory, among all of the detected pig outbreaks, a significant proportion of positive samples were recorded in the winter and summer, and mostly in those with direct contact with infected domestic pigs or wild boar. The density of the wild boar population was the most influential risk factor for the spread of ASF in wild boar living free. As the only control strategy, intensive hunting around the buffer zone may not always be sufficient to eradicate ASF [38].

ASF outbreaks among wild boar in the Baltic States and Poland can be defined as a small-scale epidemic with a slow mean spatial distribution in wild boar subpopulations (approximately 1 km/month in Lithuania and Poland to 2 km/month in Estonia and Latvia). The maximum number of positive samples was recorded when wild boar were shot in winter, which can be explained by increased hunting activity. The apparent prevalence of the virus at the national level among wild boars found dead in affected countries ranges from 60% to 86%, except for Poland, where the values range from 0.5% to 1.42% [22,39].

ASF detection methods included highly-specific real-time PCR with a universal probe library (UPL), an enzyme-linked immunosorbent assay (ELISA), and an immunoperoxidase test (IPT) to identify antibodies specific to the ASFV. Some of the countermeasures for disease prevention include the early diagnosis of ASF by identifying ASFV DNA, as well as the detection of specific antibodies by systematic serological screening [22,40]. The developed diagnostic methods allow the detection of both pathogens (ASF virus and CSF) in the same tested sample, thus saving time, labour, and money spent on studies [41]. Before the first outbreak in the EU, the infection was reported mainly on pig farms with low biosecurity and accidental contact of wild boar with domestic pigs [42].

ASF is one of the most dangerous infections that can affect pig farming due to the lack of vaccines and the serious socio-economic effect [33,43]. Therefore, early detection of the pathogen and coordinated countermeasures are urgently needed. For these countermeasures, information on the dynamics and evolution of the disease is required [7].

Prevention, early detection, a rapid response, and eradication play a crucial role in the combating of ASF. Currently, the best means of combating this disease is appropriate surveillance, which allows for disease detection in both domestic pigs and wild boar as early as possible, as well as the implementation of consolidated contingency plans in emergency situations. Classical surveillance strategies, such as active and passive surveillance on farms and slaughterhouses, targeted surveillance, together with traditional biosecurity and sanitation measures, have led to the eradication of the disease even in countries where the epidemiological role of soft ticks has been demonstrated. Historical analysis of surveillance data has shown that eradication was possible even when technological tools were missing or less widely used than today. This emphasizes that surveillance data and animal populations are crucial to planning effective monitoring and identifying appropriate control and intervention strategies [44]. There is no evidence in the scientific literature that the wild boar population in Europe can be drastically reduced by hunting or trapping. The main reasons are the adaptive behaviour of European wild boar, compensatory population growth, and the possible migration of animals from the surrounding areas. Thus, wild hunting is not an efficient tool for reducing the risk of the introduction and spread of ASF in wild boar populations. Furthermore, wild boar density thresholds for the introduction and spread of ASF in wild boar populations cannot currently be established due to uncertainty about the extent of prevalence and persistence of ASF. Attempts to drastically reduce wild boar populations may even increase transmission and contribute to the geographical spread of ASF, as intense hunting pressure on wild boar populations leads to movement of groups and individuals. Additional feeding of wild boars can increase the risk of spreading ASF. Fencing may restrict wild boar movement, but additional knowledge of the epidemiology of ASF and the spatial distribution of wild boar is needed to identify areas where fencing can be used as one of the possible elements of the eradication program [45]. In the 1970s of the 20th century, experiments were performed to reproduce the population of wild boars by feeding in the period from May to August. As a main result, the population increased almost seven times due to an increase in the number of females [46]. Measures such as attempts to reduce the wild boar population by more than 70% will theoretically be effective in combating ASF, but in practice it is impossible to achieve such an effect during a single hunting season. A combination of different tools, such as eliminating contact with carcasses and intensifying conventional hunting, reducing reproduction next year by 30–40%, could be effective in stopping the spread of ASF in wild boar [47,48]. 

Passive surveillance is the most appropriate and effective method of monitoring and the early detection of ASF in free territories. Following the focal introduction of ASF, wild boar populations should be kept at rest for a short period (e.g., ban hunting of all species, leaving unharvested crops to provide food and shelter in the affected area). The ‘white zone’ areas with a limited wild boar number may protect ASF-free areas from the disease transmission [49]. 

The proximity of some affected areas to the borders of the European Union (<150 km) has raised concerns about the possible economic consequences of ASF invasion into the EU pig sector. Establishing effective surveillance, control, and eradication programs that cover all subjects (veterinarians, farmers, and policy makers) is important in tackling ASF. Countries without ASF should be aware of the potential risk of ASF outbreak occurrence and take precautions to mitigate the risk, such as trade controls and other biosecurity measures [18,19,50]. 

Farmers do not immediately report a potential ASF outbreak, most likely due to a decline in their reputation in the local community, confidence in the self-monitoring of the outbreak without the involvement of veterinary services, and the time-consuming ASF laboratory diagnosis. The ASF control strategy should consider international experience (especially Spanish) and the local situation and be based on several important steps, including the rapid localisation of the disease by trained specialists, creation of buffer zones, continuous monitoring of pigs and farms, improvement in diagnostic tools, training of veterinary personnel, development of information systems, and international cooperation [20,21]. Recently, the role of wild boar (as a source of infection) in the occurrence and spread of ASF has been widely discussed. There are different points of view on this issue, but no final statement has been made. The above indicates the need for a more detailed study of the epizootic process, which will develop scientifically sound measures to prevent and combat this disease.

The purpose of our study was to perform a retrospective analysis of the spread of African swine fever in Ukraine in 2012–2022, to evaluate the role of wild boars in the ASF epizootic process in Ukraine.

## 2. Materials and Methods

The study was based on statistical data collected from the Department of Anti-Epizootic Measures of the Food Safety and the Veterinary Medicine Department of the Main Directorate of the State Food and Consumer Service of Ukraine, the FAO Technical Assistance Project ‘Building the potential for early detection and response to African Swine Fever in Ukraine’ www.asf.vet.ua accessed on 15 October 2022, development of ecology passports of the Ministry for protection environment and natural resources in 11 regions of Ukraine (Odessa, Poltava, Mykolaiv, Chernihiv, Rivne, Kherson, Kharkiv, Zakarpattia, Sumy, Kyiv, Donetsk), diagnostic reports of the State Research Institute for Laboratory Diagnostics and Veterinary Sanitary Examination, acts of epizootiologic investigations of veterinary departments in areas with ASF outbreaks. Data preparation, simple statistical calculations, and visualisations were prepared using Microsoft Excel software (version 2019, Microsoft, Redmond, WA, USA).

## 3. Results and Discussion 

**Figure 1:**
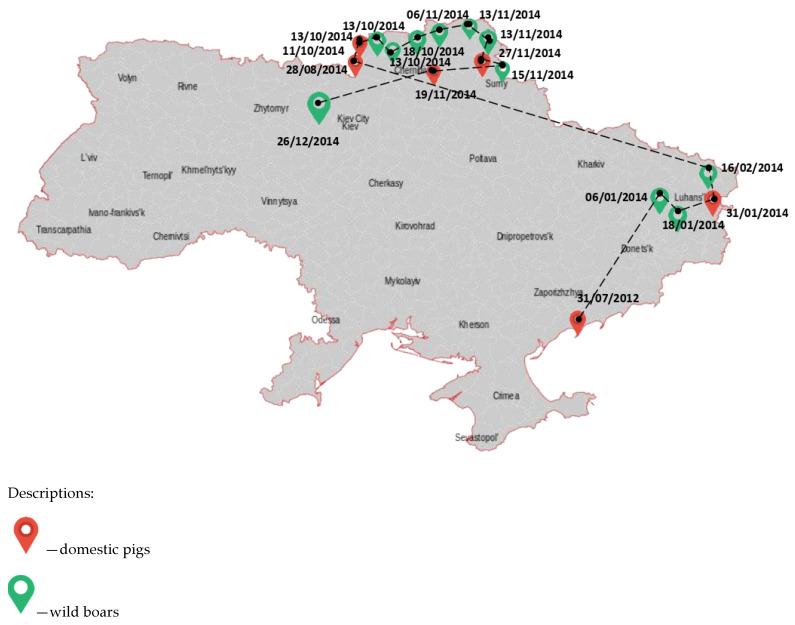
The occurrence of African swine fever in Ukraine between 2012 and 2014.

African swine fever in Ukraine was first registered in March 1977 in the Odessa region during health measures, and the epizootic was eliminated within 6 months of the primary outbreak. The cause of the outbreak was the pigswill brought by sailors arriving into the port of Odessa. The port had a subsidiary farm, and there was an infection of the pigs on this farm, which used cooking waste from the galleys of seagoing vessels to feed the pigs, including those that came to the port of Odessa from Brazil and the Dominican Republic. Then, to eliminate the virus, all pigs were destroyed. There were also two secondary outbreaks in the Kyiv and Sverdlovsk regions, where the virus was brought in by Odessa citizens through food parcels. After the elimination of these outbreaks on the territory of the former Soviet Union, no ASF outbreaks were registered in pigs. The following ASF outbreaks in Ukraine were recorded in July 2012 among pigs on a private farm in the Zaporizhian region (Figure 1).

**Figure 2:**
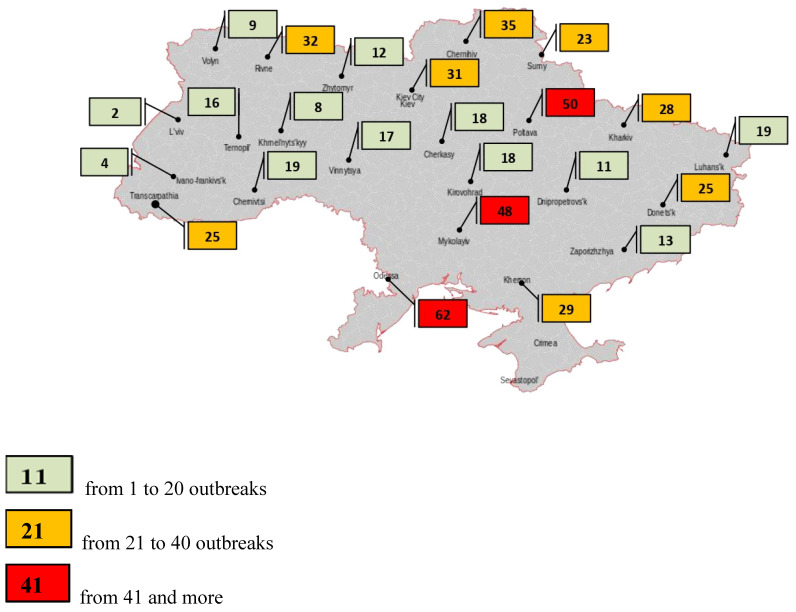
Number of African swine fever outbreaks in wild boar in Ukraine for 2012–2022.

The probable source of infection was the introduction of the pathogen by vacationers with a food of animal origin from the Russian Federation. As a result, a plan of measures was developed to locate and eliminate the outbreak of infection. The boundaries of the epizootic centre were determined as the first threat zone, within a radius of 10 km, the second covered an area of up to 150 km from the epizootic centre. Pig farms were transferred to a closed mode of operation, thus limiting the contact of pigs with other domestic animals including dogs and cats. In large farms, a mandatory clothes change was introduced as well as additional disinfection procedures. Measures taken in the Zaporizhian region were effective and allowed the elimination of the source of the disease, which did not occur during the following 17 months. It should be noted that in 2014 in the Sumy region, ASF was found in a wild boar carcass about 1.5 km from the state border with the Russian Federation. This outbreak illustrates the ability of wild boar to become the main carriers of transboundary infection and implicates a probable source of ASF. Then 12 of 16 officially confirmed ASF outbreaks were reported in the wild boar population. It is officially believed that the ASFV was transmitted by infected wild boar migrating from Belarus and Russia. The ASFV has spread uncontrollably across the territory of Ukraine. According to statistics, for the period between 2012 and 2022, the highest number of ASF outbreaks in wild boar was recorded in the Odessa (62 cases, 11.18%), Poltava (50 cases, 9%), and Mykolaiv regions (48 cases, 8.65%) of Ukraine (Table 1, Figure 2). 

Significant ASF outbreaks were reported in the Chernihiv (35 outbreaks, 6.32%), Rivne (32 outbreaks, 5.76%), Kyiv (31 outbreaks, 5.58%), Kherson (29 outbreaks, 5.22%), Kharkiv (28 outbreaks, 5.04%), Donetsk (25 outbreaks, 4.50%), and Zakarpattia (25 outbreaks, 4.50%) regions of Ukraine. A small number of outbreaks were notified in Luhansk and Chernivtsi (19 outbreaks, 3.42%, respectively), Cherkasy and Kirovohrad (18 outbreaks, 3.25%, respectively), Vinnytsia (17 outbreaks, 3.07%), Ternopil (16 outbreaks, 2.90%), Zaporizhia (13 outbreaks, 2.34%), Zhytomyr (12 outbreaks, 2.16%), and Dnipropetrovsk (11 outbreaks, 1.98%). The less affected regions included: Volyn (9 outbreaks, 1.62%), Khmelnytsky (8 outbreaks, 1.44%), Ivano-Frankivsk (4 outbreaks, 0.72%), and Lviv (2 outbreaks, 0.37%).

Most of the ASF outbreaks in wild boar that were registered in Ukraine in 2012–2014 were identified between the autumn and winter period. 

Between 2012 and 2013, most ASF outbreaks in wild boar on Ukrainian territory were detected within the afforested area. Meanwhile, between 2014 and 2016, ASF was transmitted to backyard farms. The outbreaks in backyards were registered as a series of ‘waves’ that occurred over a period of 13 to 30 days. Similarly, in 2015, about 40 ASF outbreaks were registered on domestic pig farms. The most important factor responsible for the transmission of ASF in Ukraine between backyard farms was human activity. Between 2017 and 2018, ASF outbreaks in domestic pigs were reported in Ukraine every two days.

**Figure 3:**
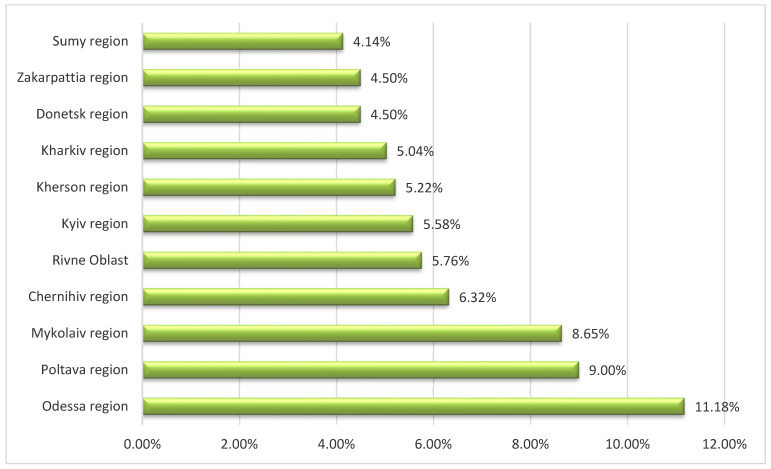
The occurrence of African swine fever outbreaks in the domestic pig sector among 11 regions of Ukraine mostly affected between 2012 and 2022.

Among all ASF-affected regions of Ukraine, the most frequent occurrence of the disease has been observed in three areas, namely, Odessa, Poltava, and Mykolaiv (Figure 3). In our opinion, there are several reasons causing the high prevalence of ASF outbreaks, including the lack of health certificates for domestic pigs, illegal pig movements, and uncontrolled trade of pigs and pig products.

**Figure 4:**
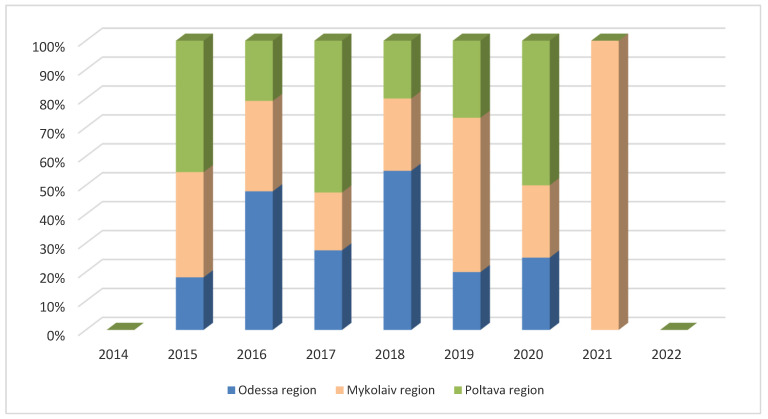
Dynamics of the occurrence of African swine fever outbreaks in domestic pigs in the most affected regions of Ukraine between 2012 and 2022.

A significant proportion of African swine fever outbreaks were recorded in the Odessa region between 2016 and 2018, as well as in the Poltava region in 2015, 2017, and 2020, as well as in the Mykolaiv region in 2019 and 2021 (Figure 4).

The emergence of ASF in Ukraine in pig production has been observed as ‘waves’ of epizootics present during the following time intervals. (Table 2).

**Figure 5:**
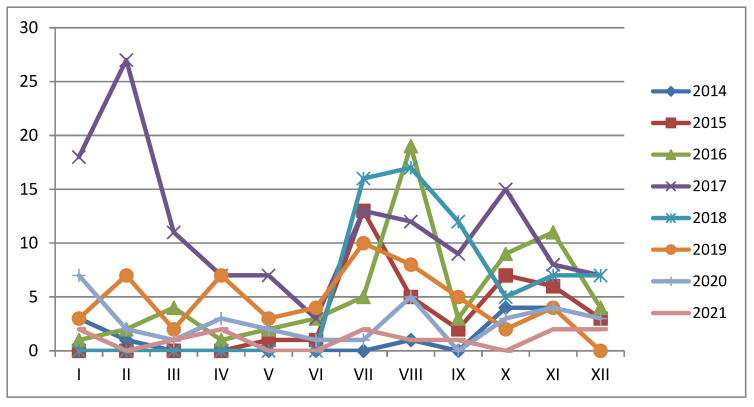
Seasonality of African swine fever outbreaks among domestic pigs between 2014 and 2021.

It has been observed that ASF in Ukraine is characterised by seasonality (Figure 5), with the highest outbreak incidence observed between summer and autumn, except in 2017 when the highest number of outbreaks was registered in winter. The potential sources of infection may be related to the increased activity of farmers and the application of agricultural machines in the area of the ASFV’s presence in contaminated fields. Others might be related to the application of ASFV-contaminated feed from not registered ASF outbreaks.

Analysing the data, it was found that the highest number of outbreaks in domestic pigs was observed in pig farms (more than 50%), showing a low level of biosecurity and a lack of awareness of the owner in the context of the threat of ASF.

A significant role in the spread of ASF was played by the improper disposal of domestic pigs from ASF outbreaks by dumping dead animals in landfills and forests. This may be the main reason for the uncontrolled circulation of the ASFV between the population of dead pigs and wild boar. Another threat was the long-term storage of ASFV-contaminated meat in slaughterhouses with their parallel illegal trade. This may also be a reason for the unexpected emergence of ASF in different regions of Ukraine.

It was difficult to assess the current prevalence of ASF among wild boar due to the absence of the possibility of performing an adequate analysis of the epizootic situation. The main constraint was represented by the lack of representative data focused on passive and active ASF surveillance. It should be mentioned that very little is known about the pathogenicity of the current circulating ASFV strains in the wild boar population, as well as the mortality caused by ASF observed among infected animals. 

**Figure 6:**
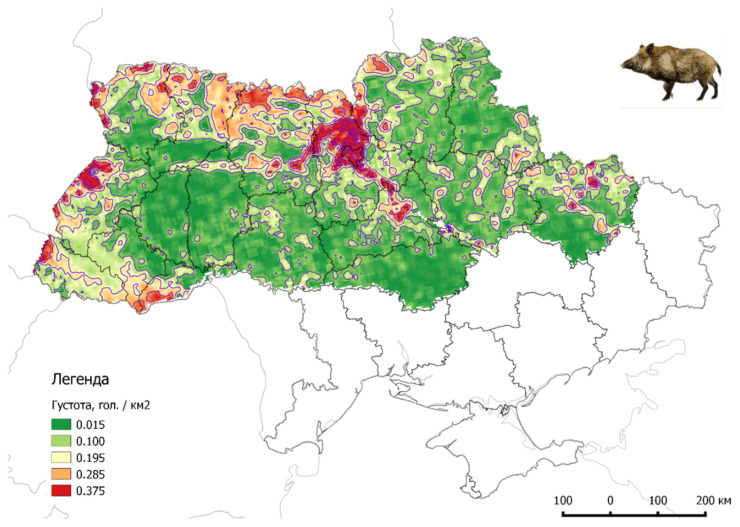
Density of the wild boar population on Ukrainian territory (2022, source: www.asf.vet.ua accessed on 15 October 2022).

The habitat of wild boar in Ukraine is significant, but most are settled in the northern and western regions, with a population density ranging from 0.02 to 0.50 wild boar/km^2^. In some areas in northern Ukraine, the population density reaches even 8 wild boars/km^2^ (Figure 6).

**Figure 7:**
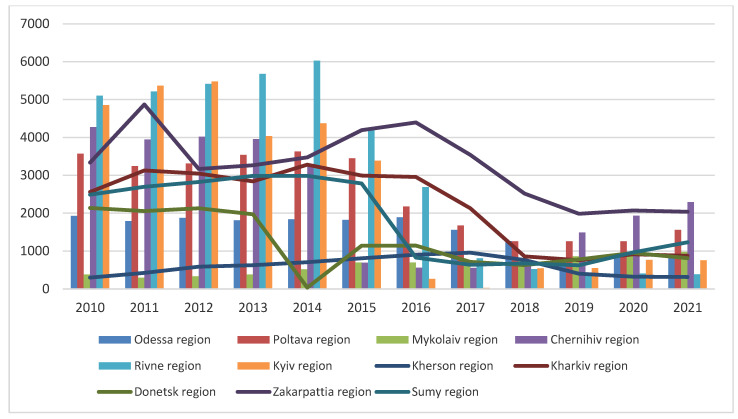
Wild boar population in some regions of Ukraine in 2010–2020 according to the data from hunting grounds (hunting bag).

The efforts to limit the wild boar population in Ukraine in particular hunting grounds between 2010 and 2021 are shown in Figure 7 Consequently, according to the ‘hunting bag’ data, a significant decrease in the number of wild boars was observed in 2018, by 24.9%; in 2017, by 30.1%; and in 2016, by 36.9%.

**Figure 8:**
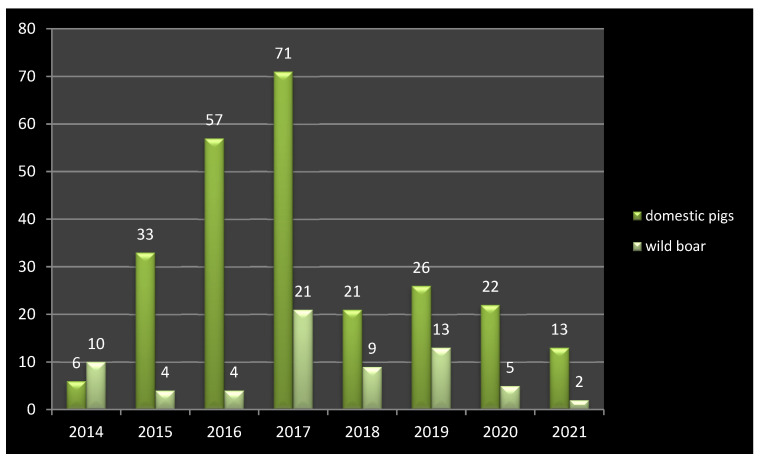
ASF outbreaks in Ukraine between 2014 and 2021 in domestic pigs and wild boar.

During hunting seasons in Ukraine, 6.5 to 7 thousand wild boars are shot, but only 1.5 to 2 thousand are tested for the ASFV due to the ASF surveillance funding constraints (Figure 8). The highest number of ASF outbreaks in wild boar was observed in 2019 (13 outbreaks), while in domestic pigs this was in 2017 (71 outbreaks). On the other hand, we believe that the reduction in the wild boar population to the expected limit of 0.4 wild boar/km² will not stop the further expansion of ASF.

## 4. Conclusions

For most of the area of Ukraine, the prevalence of ASF in wild boar populations probably depends largely on the density of these animals. The epizootic chain between the infected wild boar population and backyard farms with a low level of biosecurity complicates the efforts to eradicate ASF in Ukraine. One of the most important factors of the ASFV spread between farms is represented by contaminated feed, as well as the illegal trade of pigs. The lack of awareness among pig farmers causes the improper disposal of pigs from ASF outbreaks. In conclusion, as a result of all the mentioned risk factors, sporadic ASF outbreaks should be expected in Ukrainian pig production in the future. The additional and major issue with a huge negative impact on Ukrainian pig production is caused by the Russian offensive.

A trend of a gradual annual decrease in the number of ASF outbreaks in the wild boar population and domestic pigs has been observed. However, this may also be caused by limitations in the continuous passive and active surveillance in the wild boar population in Ukraine. An improved strategy to monitor the epizootic situation and optimise further measures to combat ASF should be considered and implemented. The lack of an effective vaccine or treatment, with no universal measures of the eradication of ASF, complicates further efforts to protect the Ukrainian pig production sector from this disease. 

The results of ASF surveillance in Ukraine indicate the circulation of the virus between the infected wild boar population and the backyard and non-commercial sector of pig production. This confirms the non-compliance with biosecurity rules and the lack of awareness among pig producers. 

Due to the circulation of the ASFV in the wild boar population, it is necessary to rebuild the pig production system in Ukraine from backyard farms to well-protected commercial farms in order to stop the further expansion of ASF to neighbouring countries. Hopefully, these plans will be implemented after the still ongoing Russian invasion of Ukraine is finished. 

## Figures and Tables

**Table 1:** Dynamics of African Swine Fever Spread in Wild Boar Population in Ukraine between 2012 and 2022.

Region	2012	2014	2015	2016	2017	2018	2019	2020	2021	2022	In General	%
Vinnytsia Region	-	-	-	1	8	2	3	2	1	-	17	3.07
Volyn region	-	-	-	4	1	1	2	1	-	-	9	1.62
Dnipropetrovsk Region	-	-	-	-	4	2	3	2	-	-	11	1.98
Donetsk Region	-	-	-	-	7	12	5	1	-	-	25	4.50
Zhytomyr Region	-	-	1	2	1	3	3	1	-	1	12	2.16
Zakarpattia region	-	-	-	1	13	11	-	-	-	-	25	4.50
Zaporizhzhian Region	1	-	-	-	4	3	3	1	1	-	13	2.34
Ivano-Frankivsk Region	-	-	-	-	3	-	-	1	-	-	4	0.72
Kyiv City	-	-	-	-	-	-	-	-	1	-	1	0.18
Kyiv region	-	-	6	2	7	10	2	3	-	1	31	5.58
Kirovohrad Region	-	-	-	5	7	-	4	1	1	1	19	3.24
Luhansk Region	-	4	-	-	10	4	-	-	1	-	19	3.42
Lviv Region	-	-	-	-	1	-	1	-	-	-	2	0.37
Mykolaiv region	-	-	4	15	8	10	8	1	2	-	48	8.65
Odessa Region	-	-	2	23	11	22	3	1	-	-	62	11.18
Poltava Region	-	-	5	10	21	8	4	2	-	-	50	9.00
Rivne Region	-	-	2	4	12	13	1	-	-	-	32	5.76
Sumy Region	-	1	6	5	2	6	1	2	-	-	23	4.14
Ternopil Region	-	-	-	-	5	6	3	2	-	-	16	2.90
Kharkiv Region	-	-	-	8	17	-	2	-	1	-	28	5.04
Kherson Region	-	-	-	-	9	13	3	1	3	-	29	5.22
Khmelnytskyi Region	-	-	-	5	1	1	1	-	-	-	8	1.44
Cherkasy Region	-	-	1	3	6	8	-	-	-	-	18	3.25
Chernivtsi Region	-	-	-	1	2	7	3	1	5	-	19	3.42
Chernihiv Region	-	11	13	2	3	3	-	2	-	1	35	6.32
Total	1	16	40	91	163	145	55	25	16	4	556	100

**Table 2:** The ‘waves’ of ASF epizootic in Ukraine (1997–2022).

Months	Years											
	1997	2012	2013	2014	2015	2016	2017	2018	2019	2020	2021	2022
I												
II												
III												
IV												
V												
VI												
VII												
VIII												
IX												
X												
XI												
XII												

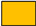
 Note: the presence of outbreaks of African swine fever in domestic pigs.

## Data Availability

Not applicable.
